# Unraveling implicit human behavioral effects on dynamic characteristics of Covid-19 daily infection rates in Taiwan

**DOI:** 10.1371/journal.pone.0298049

**Published:** 2024-02-12

**Authors:** Ting-Li Chen, Elizabeth P. Chou, Min-Yi Chen, Fushing Hsieh

**Affiliations:** 1 Institute of Statistical Science, Academia Sinica, Taipei, Taiwan; 2 Department of Statistics, National Chengchi University, Taipei, Taiwan; 3 Department of Statistics, University of California at Davis, Davis, CA, United States of America; National Taiwan University, TAIWAN

## Abstract

We investigate the dynamic characteristics of Covid-19 daily infection rates in Taiwan during its initial surge period, focusing on 79 districts within the seven largest cities. By employing computational techniques, we extract 18 features from each district-specific curve, transforming unstructured data into structured data. Our analysis reveals distinct patterns of asymmetric growth and decline among the curves. Utilizing theoretical information measurements such as conditional entropy and mutual information, we identify major factors of order-1 and order-2 that influence the peak value and curvature at the peak of the curves, crucial features characterizing the infection rates. Additionally, we examine the impact of geographic and socioeconomic factors on the curves by encoding each of the 79 districts with two binary characteristics: North-vs-South and Urban-vs-Suburban. Furthermore, leveraging this data-driven understanding at the district level, we explore the fine-scale behavioral effects on disease spread by examining the similarity among 96 age-group-specific curves within urban districts of Taipei and suburban districts of New Taipei City, which collectively represent a substantial portion of the nation’s population. Our findings highlight the implicit influence of human behaviors related to living, traveling, and working on the dynamics of Covid-19 transmission in Taiwan.

## 1 Introduction

The emergence and global spread of coronavirus disease 2019 (COVID-19), caused by the novel severe acute respiratory syndrome coronavirus 2 (SARS-CoV-2), has evolved into a major public health crisis. This pandemic has resulted in a sudden and overwhelming strain on hospitals, leading to an increased incidence of pneumonia with multi-organ disease and millions of fatalities in numerous countries worldwide [1, 2]. Moreover, over the past three years, COVID-19 has inflicted immeasurable economic losses upon nearly all nations, along with psychological and financial hardships experienced by billions of individuals [3–5]. However, existing literature on COVID-19 lacks a comprehensive understanding of the spreading dynamics of this infectious disease at a scale beyond private homes, crowded restaurants, or concert halls, such as within specific districts of a city. Analyzing this scale provides a certain level of homogeneity from both geographical and socioeconomic perspectives, which facilitates comparison to larger city or country scales. Although some studies have assessed the effects of model-based emergency containment measures and non-pharmaceutical interventions at this district scale [6–9], very few investigations have delved into the intrinsic nature of the spreading dynamics, except for a limited number of studies [10]. Therefore, there is a crucial knowledge gap in understanding the interplay between the spreading dynamics of infectious diseases and human geographic and socioeconomic dynamics.

To fill the knowledge gap in COVID-19 literature, this paper focuses on studying the disease’s spreading dynamics within Taiwan’s unique context. Taiwan’s distinctive role in the pandemic stems from its island status and the strict policies implemented to regulate the entry of foreigners, effectively creating a closed domain for this infectious disease. This closed system provides an opportunity to investigate the spreading dynamics within a relatively homogeneous and controlled environment. By unraveling the implicit human behaviors and their interplay with the geographic and social constituents, this study aims to identify and understand the intricate mechanisms underlying the pandemic crisis in Taiwan. This research sheds light on the importance of exploring uncharacterized and unobserved human behaviors that may have played a crucial role in shaping the spread of the disease within this special and significant context.

**Fig 1:**
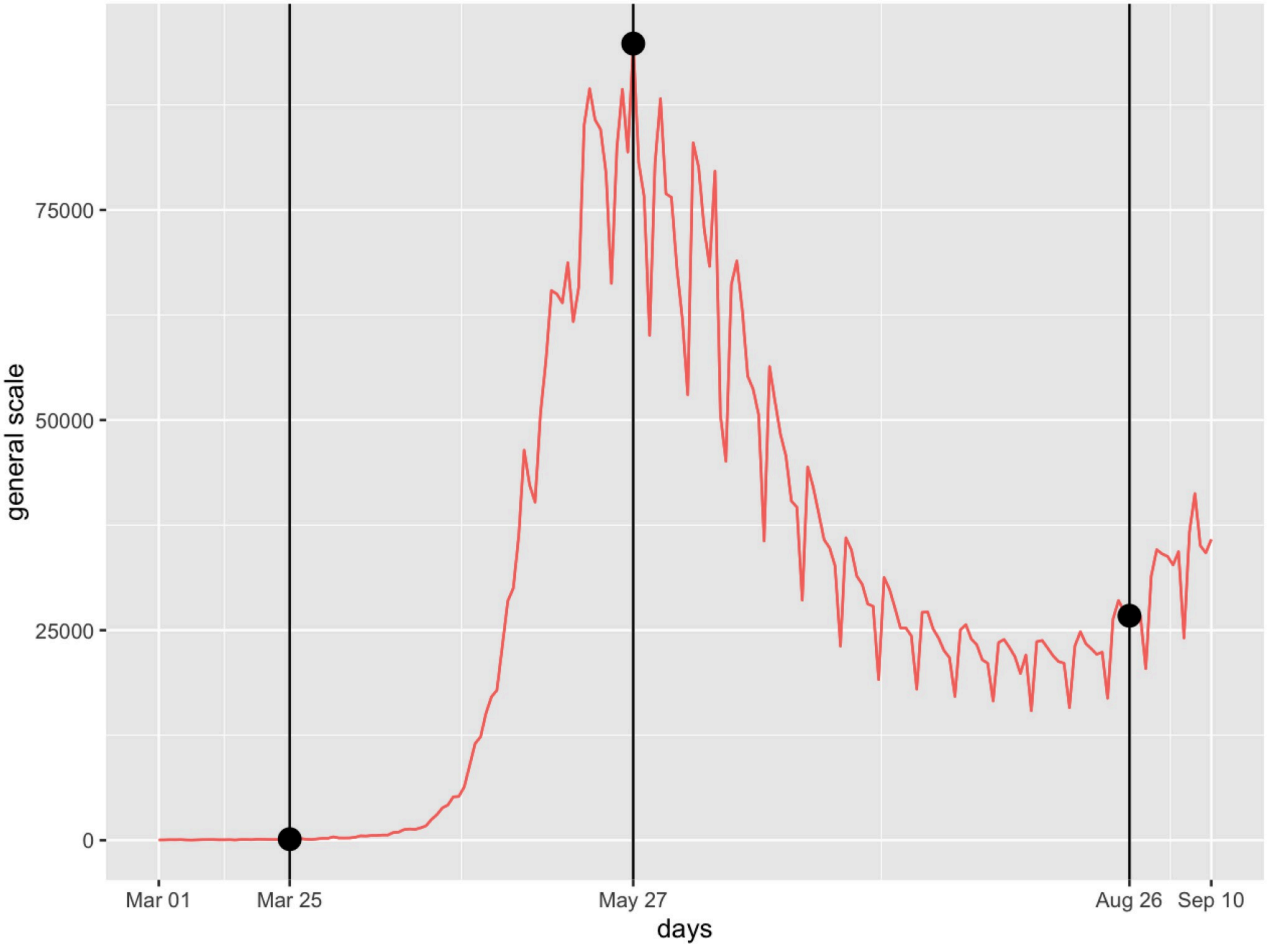
Taiwan’s curve of the daily infection rate from March to September 2022.

The time period chosen for investigation spans from March 25 to August 19, 2022, aligning with the emergence and prevalence of the Omicron variant (specifically, the ba.1 or ba.2 sublineage) in Taiwan. During this period, there was a significant upsurge in the number of COVID-19 cases compared to previous phases of the pandemic. The selection of this specific timeframe is driven by the need to understand the distinct dynamics and implications of the Omicron variant in Taiwan’s fight against the disease. The Omicron variant is known for its heightened transmissibility and potential evasion of immunity, contributing to the rapid escalation of infection rates during this period. To illustrate the trend, Fig 1 displays Taiwan’s daily infection rate curve from March to September 2022, calculated as the daily reported new cases divided by the population.

In order to comprehensively understand human behavioral patterns in the context of the COVID-19 pandemic, it is crucial to analyze data that reflects the entire population. However, obtaining publicly available human behavioral data for Taiwan’s population of 23 million is currently not feasible. Given the susceptibility of the entire population to the infectious disease, collecting individual-level behavioral data on such a large scale remains a challenge. Therefore, to gain insights into behavioral dynamics, we adopt an alternative approach by extracting behavior-related information from geographic locations and social-economic statuses. In the first example, we examine the distinction between northern and southern cities in Taiwan. The six municipalities in Taiwan are divided into two groups: Taipei, New Taipei, Taoyuan, and Keelung, considered northern cities. Keelung, although not a municipality itself, is closely situated to the other three municipalities in northern Taiwan and has played a significant role in the initial spread of infections. The remaining three cities, Taichung, Tainan, and Kaohsiung, are categorized as southern cities. By comparing the dynamics between these two regions, we aim to shed light on potential behavioral differences driven by geographic locations and their impact on the spread of COVID-19. In the second example, we investigate the influence of social-economic statuses by focusing on Taipei as an urban city and its surrounding cities, namely New Taipei, Taoyuan, and Keelung, which are considered suburban areas. Taipei, as a city that houses a majority of government branches and large business corporations, exhibits a higher overall social-economic status compared to the surrounding cities. By examining these social-economic differences, we aim to uncover potential behavioral effects induced by variations in social-economic factors.

The development of a simple computational framework is a crucial component of this paper, as it enables the study of human behavioral patterns in the context of infectious disease spreading dynamics. This framework is specifically designed to fill the gap in analytical methodologies for analyzing population-wide spreading dynamics based on the curves of daily infection rates observed in various administrative districts. The unstructured nature of curve data presents a significant challenge in data analysis. To overcome this challenge, our framework employs a comprehensive set of measurable features extracted from each curve. These features are carefully selected to capture the growth and decline patterns embedded within each curve. By utilizing this approach, we represent each curve with a structured vector, consisting of measurements of these features. This structured representation allows for more effective analysis and characterization of the dynamics associated with the spread of infectious diseases.

In Section 2, we provide information on where to access the data used in this study. We then outline the procedure for transforming the original data into curves representing the daily infection rate and further converting these curves into structured data in Section 3. With this structured data, we introduce a major factor selection protocol designed to identify important features that capture the individual dynamics of response features, including peak-value and curvature-at-peak. Major factors refer to a feature set that explains a significant proportion of the uncertainty in the response variable. This data-driven computational protocol, based on Theoretical Information Measurements such as conditional entropy and mutual information [11], has been developed in previous works [12–15]. We will provide a brief introduction to these key ideas in Section 4.

Our analysis in this study is structured as follows. In Section 5, we examine the associations between variables using Theoretic Information and present them through network figures. Section 6 focuses on the application of the major factor selection protocol to identify significant factors related to peak-value and curvature-at-peak. Next, in Section 7, we investigate whether the growth and decline patterns of daily infections exhibit geographic and social-economic differences. We then delve into a more detailed analysis in Section 8, exploring the effects of geography and social-economic factors at the district level and within different age groups. Finally, our study concludes with remarks and findings in Section 9.

## 2 Data

The data used in this study comprises the daily COVID-19 case counts in Taiwan, which are obtained from the Taiwan Centers for Disease Control (CDC). This data is publicly available and can be downloaded from the following website: https://covid-19.nchc.org.tw/index.php?language=en.

Population information data is sourced from the Department of Household Registration, Ministry of the Interior, Taiwan. This data is also publicly available and can be downloaded from the following website: https://www.ris.gov.tw/app/portal/346.

For transparency and reproducibility, all the data and codes used in this study can be accessed on the following GitHub repository: https://github.com/CSDA2023/covid19.

It is important to note that all data used in this study does not contain or involve any forms of information related to personal identification.

## 3 Data preprocessing

The data preprocessing stage is crucial for extracting relevant information from the unstructured curves of daily infection rate in administrative districts. These curves, resembling continuous functions or images, hide essential details related to the growth and decline of COVID-19 cases. In this section, we present our approach to extracting this pertinent information from each curve. The procedure begins by identifying measurable characteristics within the curve and computing characteristic-specific features. Each feature represents a measurement of a specific characteristic, encoding a distinct aspect of the curve’s information. By selecting a set of these features, we aim to capture the intrinsic information embedded within the curves.

To begin the data preprocessing stage, we represent a curve of daily infection rate as a time series denoted as *X*_*t*_, where *t* represents the day-axis. Our initial step involves smoothing the curve. As depicted in Fig 1, the daily reported infection cases exhibit a recurring weekly pattern due to the weekend hospital closures. Therefore, it is appropriate to apply a 7-day moving average to remove this weekly pattern. Additionally, a second 7-day moving average is employed, as the resulting curves may not consistently achieve the desired level of smoothness. Mathematically, applying a 7-day moving average twice is equivalent to a 13-day weighted average, expressed as:

$${\tilde{X}}_{t}=\sum_{d=-6}^{6}\left(\frac{7+d}{49}\right)\times X_{t+d}.$$

**Fig 2:**
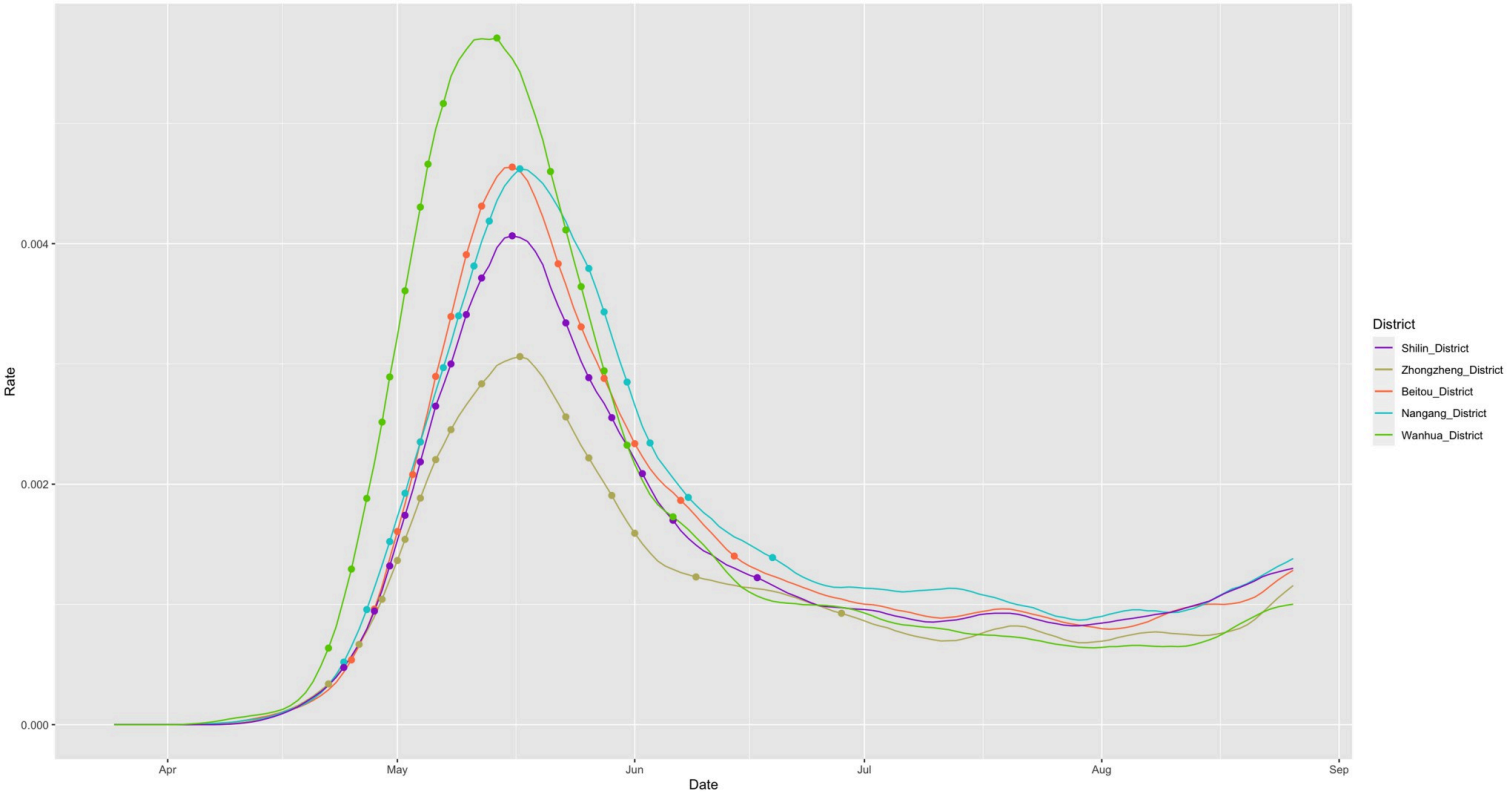
Five smoothed curves of Taipei’s 12 districts.

By applying this smoothing technique, we aim to achieve the desired level of smoothness while effectively capturing the unique growth and decline patterns within the curve. Empirical observations indicate that the trajectory of ${\tilde{X}}_{t}$ generally exhibits strict growth leading up to a distinct peak, followed by a steady decline until the end of the study period on August 19. Fig 2 provides an illustrative example showcasing the smoothed curves of daily infection rate for five districts in Taipei. Each curve exhibits a single mode, with sharp growth on the left side of the peak and a more gradual decline on the right side. These uni-modal patterns, characterized by sharp growth and gentle decline, serve as defining characteristics of the curves. In this section, we will select and extract features that collectively capture these characteristic patterns.

Based on the smoothed curve ${\tilde{X}}_{t}$, we extract two peak-related features. Firstly, we identify the date of the “peak” as *t*_*max*_, which corresponds to the day-*t* when ${\tilde{X}}_{t}$ reaches its maximum value. Secondly, we extract the “peak value” denoted as ${\tilde{X}}_{t_{max}}$. Additionally, we determine the first time before *t*_*max*_ when ${\tilde{X}}_{t}$ surpasses 90% of ${\tilde{X}}_{t_{max}}$ during its growth phase. We denote this feature as *t*_−0.1_, and it is defined as follows:

$$t_{-0.1}=\text{inf}\left\{t|{\tilde{X}}_{t}\geq 0.9\times {\tilde{X}}_{t_{max}}\right\}.$$

The negative sign in the subscript indicates that it is located to the left of *t*_*max*_. Conversely, we define *t*_0.1_ as the last time after *t*_*max*_ when ${\tilde{X}}_{t}$ remains above 90% of ${\tilde{X}}_{t_{max}}$:

$$t_{0.1}=\text{sup}\left\{t>t_{max}|\underset{s\geq t}{\text{sup}}{\tilde{X}}_{s}\geq 0.9\times {\tilde{X}}_{t_{max}}\right\}.$$

Likewise, we extract 7 features *t*_−0.8_, *t*_−0.7_, …, *t*_−0.2_ on the left of *t*_−0.1_ and another 7 features *t*_0.2_, *t*_0.3_, …, *t*_0.8_ on the right of *t*_max_ as well. Their precise definitions are given as follows: for 0.2 ≤ *α* ≤ 0.8,

$$\begin{array}{ccc} t_{-\alpha } & = & \text{inf}\{t<t_{max}|{\tilde{X}}_{t}\geq \left(1-\alpha \right)\times {\tilde{X}}_{t_{max}}\},\\ t_{\alpha } & = & \text{sup}\{t>t_{max}|\underset{s\geq t}{\text{sup}}{\tilde{X}}_{s}\geq \left(1-\alpha \right)\times {\tilde{X}}_{t_{max}}\}. \end{array}$$

With the information derived from these 18 features of ${\tilde{X}}_{t}$, we can reconstruct ${\tilde{X}}_{t}$ with relatively high precision. For example, we can evaluate the positive slope of ${\tilde{X}}_{t}$ on the interval (*t*_−0.3_, *t*_−0.2_) and that on the interval (*t*_0.2_, *t*_0.3_), among others. However, we do not extract *t*_−0.10_ due to stability concerns, while *t*_0.10_ has not yet been reached by any districts.

**Fig 3:**
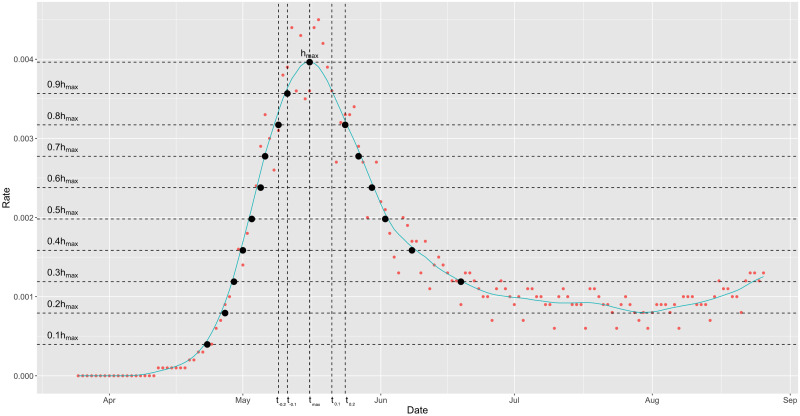
Illustrative definitions and extractions of all features. The red dots represent the original data points *X*_*i*_, while the black dots represent the corresponding values of *t*_*α*_.

In addition to the 18 features mentioned earlier, we derive two calculated features as follows. The first calculated feature is the “robust-peak” *t*_0_, which is defined as the largest integer not exceeding the middle point of the interval [*t*_−0.1_, *t*_0.1_]. In most districts, *t*_0_ and *t*_*max*_ are nearly equal. However, due to the observed asymmetry in the growth and decline of ${\tilde{X}}_{t}$, it is evident that *t*_0_ ≥ *t*_*max*_. The second calculated feature is the “curvature-at-peak,” which is defined as the length of the interval [*t*_−0.1_, *t*_0.1_]. This feature provides insight into the curvature of ${\tilde{X}}_{t}$ at *t*_0_. These calculated features play a crucial role in defining response variables and investigating the underlying dynamics of the formation of curves of daily infection rates. For illustrative examples of these defined quantities, refer to Fig 3.

To enable a meaningful and comparative analysis of the shapes of all curves of daily infection rates, we define several variables based on the previously defined 18 features. These variables include the “peak” feature, calculated as *t*_*max*_−*t*_0_, the “peakvalue” feature denoted as ${\tilde{X}}_{t_{max}}$, the “left90” feature represented by *t*_0_ − *t*_−0.1_, the “right90” feature indicated by *t*_0.1_ − *t*_0_, the “left80” feature calculated as *t*_0_ − *t*_−0.2_, the “right80” feature defined as *t*_0.2_ − *t*_0_, and so on. By utilizing these variables, we eliminate the calendar coordinate information from the features *t*_*α*_ for −0.8 ≤ *α* ≤ 0.8 and retain only their shape information. Consequently, we can compare the shapes of two curves of daily infection rates by examining the values of the 18 feature variables: peak, peakvalue, left90, left80, …, left20, right90, right80, …, right20 as if they were aligned with a common *t*_0_. The only feature variable that retains the calendar coordinate is the “peakdate” feature, represented by *t*_max_.

Measurements of these 18 feature variables across all potential district-specific or age-group-specific curves are collected from 7 cities under study in Taiwan. These measurements are of discrete data type and are stored in a structured data matrix format, where each row represents a curve and each column represents a feature variable. It is important to note that these feature variables retain their mutual associations of various degrees. The variations in the degrees of association are expected to be significantly heterogeneous. For instance, the feature variables on the left, depicting growth patterns, are expected to be highly associated, while the feature variables on the right, depicting decline patterns, are less so. This asymmetry in association reveals their essential roles, among many other distinct roles, in characterizing the dynamics underlying curves of daily infection rates, as discussed in Section 5.

## 4 Basic methods

In this section, we utilize the structured data matrix, consisting of measurements of the 18 selected features, to introduce computational concepts and methodologies based on Theoretical Information Measurement ([11]) that are necessary for the data analysis presented in this paper. In the first subsection, we provide a brief review of the concepts of conditional entropy and mutual information. The concept of conditional entropy will be employed to establish directed and undirected association measurements between two features, as well as between two feature sets of different sizes. In the second subsection, we provide a concise overview of the major factor selection protocol used to examine the dynamics of a chosen response variable with respect to a set of covariate features. This protocol, developed in [12–15], builds upon the concepts of conditional entropy and mutual information.

### 4.1 Association based on theoretic information

In this paper, we analyze the association between two variables based on Theoretic Information. There are various statistics that can measure such association, and in our analysis, we employ Theoretic Information as the basis. The entropy of a variable *X* is defined as $H\left(X\right)=E_{p}\left[-\text{log}\left(p\left(X\right)\right)\right]$, where *p*(*X*) represents the probability distribution of *X*. This entropy value represents the amount of information or uncertainty associated with the variable *X*. To investigate the association between two variables *X* and *Y*, we can examine their conditional distributions *X*|*Y*, *Y*|*X*, or the joint distribution (*X*, *Y*). Through these distributions, we can quantify the association between *X* and *Y* as

$$\begin{array}{c} H(X,Y)=H(X)+H(Y|X) \end{array}$$ (1)

$$\begin{array}{c} =H(X|Y)+H(Y) \end{array}$$ (2)

$$\begin{array}{c} =H(X|Y)+I(X,Y)+H(Y|X), \end{array}$$ (3)

where

I(X,Y)=H(X)-H(X|Y)=H(Y)-H(Y|X)

is the mutual information of *X* and *Y*. The conditional entropy *H*(*X*|*Y*) measures the uncertainty of *X* given the knowledge of *Y*, or equivalently, the information in *X* that is not explained by *Y*. To quantify the ratio of information in *X* that is not explained by *Y*, we define the re-scaled conditional entropy as

$$E\left(X|Y\right)=\frac{H(X|Y)}{H(X)}.$$

This measure ranges from 0 to 1, where E(X|Y)=0 indicates that *Y* can perfectly explain *X*, while E(X|Y)=1 indicates that knowing *Y* provides no information about *X*.

Using this statistic, we construct a network graph that represents the associations between variables. Each variable corresponds to a vertex in the graph, and an edge is drawn between two variables if their re-scaled conditional entropy is below a specified threshold. The width of the edge is used to indicate the strength of the association between the two variables.

In addition to the network graph, we also visualize the associations between variables using a heatmap. The heatmap displays the re-scaled conditional entropies between variables. To enhance the interpretability, we rearrange the rows and columns of the heatmap based on a Hierarchical Clustering Tree, which helps reveal the underlying structures of the variables.

### 4.2 Major factor selection

In the previous subsection, we discussed how the conditional entropy *H*(*Y*|*X*) represents the information of the response variable *Y* not yet explained by the covariate variable *X*. When *Y* is the response variable of interest, a lower value of *H*(*Y*|*X*) indicates that *X* plays a more crucial role in explaining *Y*. Our objective in this context is to identify a subset $F\subset X_{1},X_{2},\ldots ,X_{p}$ such that the conditional entropy H(Y|F) is minimized. F represents the set of feature variables that serve as the major factors, as named in our previous work, in explaining the dynamics between the response variable *Y* and the covariates.

In our previous work [12], we introduced a protocol for major factor selection. The algorithm begins by examining the individual effect of each covariate by computing the conditional entropies. Covariates that exhibit significantly lower conditional entropy are identified as potential candidates for the major factors influencing the dynamics between the response variable and the covariates (Re-Co dynamics).

Next, we proceed to calculate the conditional entropies for every pair of covariates. In theory, we expect that *H*(*Y*|*X*_*i*_) ≥ *H*(*Y*|*X*_*i*_, *X*_*j*_), and in practice, this inequality is typically strict due to the finite sample phenomenon. This suggests that adding another covariate to a feature set can improve the explanation of the response variable. However, it is important to ensure that the newly added covariate provides a significant improvement beyond independent noise. To address this, we propose a fast algorithm to estimate the reduction in entropy caused by noise, which serves as a standard for determining the significance of the entropy drop.

In addition to identifying redundant covariates in a feature set, we also examine the 2nd-order effect, which arises when both covariates are present together. This occurs when the 2-feature effect is significantly larger than the sum of the individual 1-feature effects. The 2-feature effect is quantified by *H*(*Y*) − *H*(*Y*|*X*_*i*_, *X*_*j*_), while the individual 1-feature effects are given by *H*(*Y*) − *H*(*Y*|*X*_*i*_) and *H*(*Y*) − *H*(*Y*|*X*_*j*_), respectively. To check

$$H\left(Y\right)-H\left(Y|X_{i},X_{j}\right)\gg H\left(Y\right)-H\left(Y|X_{i}\right)+H\left(Y\right)-H\left(Y|X_{j}\right),$$

it suffices to check

$$H\left(Y|X_{i}\right)-H\left(Y|X_{i},X_{j}\right)\gg H\left(Y\right)-H\left(Y|X_{j}\right).$$

Successive conditional entropy (SCE) drop is defined as

$$\text{SCE-drop}\left(F\right)=\underset{X_{i}\in F}{\text{min}}H\left(Y|F\backslash X_{i}\right)-H\left(Y|F\right).$$

Therefore, we check whether the 2-order effect exists from

$$\text{SCE-drop}\left(X_{i},X_{j}\right)\gg \text{min}\left\{\text{SCE-drop}\left(X_{i}\right),\text{SCE-drop}\left(X_{j}\right)\right\}.$$

Higher-order effects are checked by similar formulas.

## 5 Associative relationships among all feature-variables

In this section, we investigate the associative relationships among the 18 feature variables, which are designed to capture the growth and decline patterns observed in the curves of daily infection rates over a fixed study period. These associations are visually demonstrated in Fig 3 and can also be observed in Fig 2. To further examine and quantify these associations, we utilize measurements such as conditional entropy and mutual information.

We begin by categorizing each of the 18 feature variables into four categories. This categorization serves two purposes: reducing noise and enabling the detection of potentially nonlinear associative patterns using contingency tables. Specifically, we construct a 4 × 4 contingency table for each pair of these 18 features. Using this contingency table framework, as explained in Section 4, we calculate the re-scaled conditional entropy to assess the directional associations of row-variable-to-column-variable and column-variable-to-row-variable.

**Fig 4:**
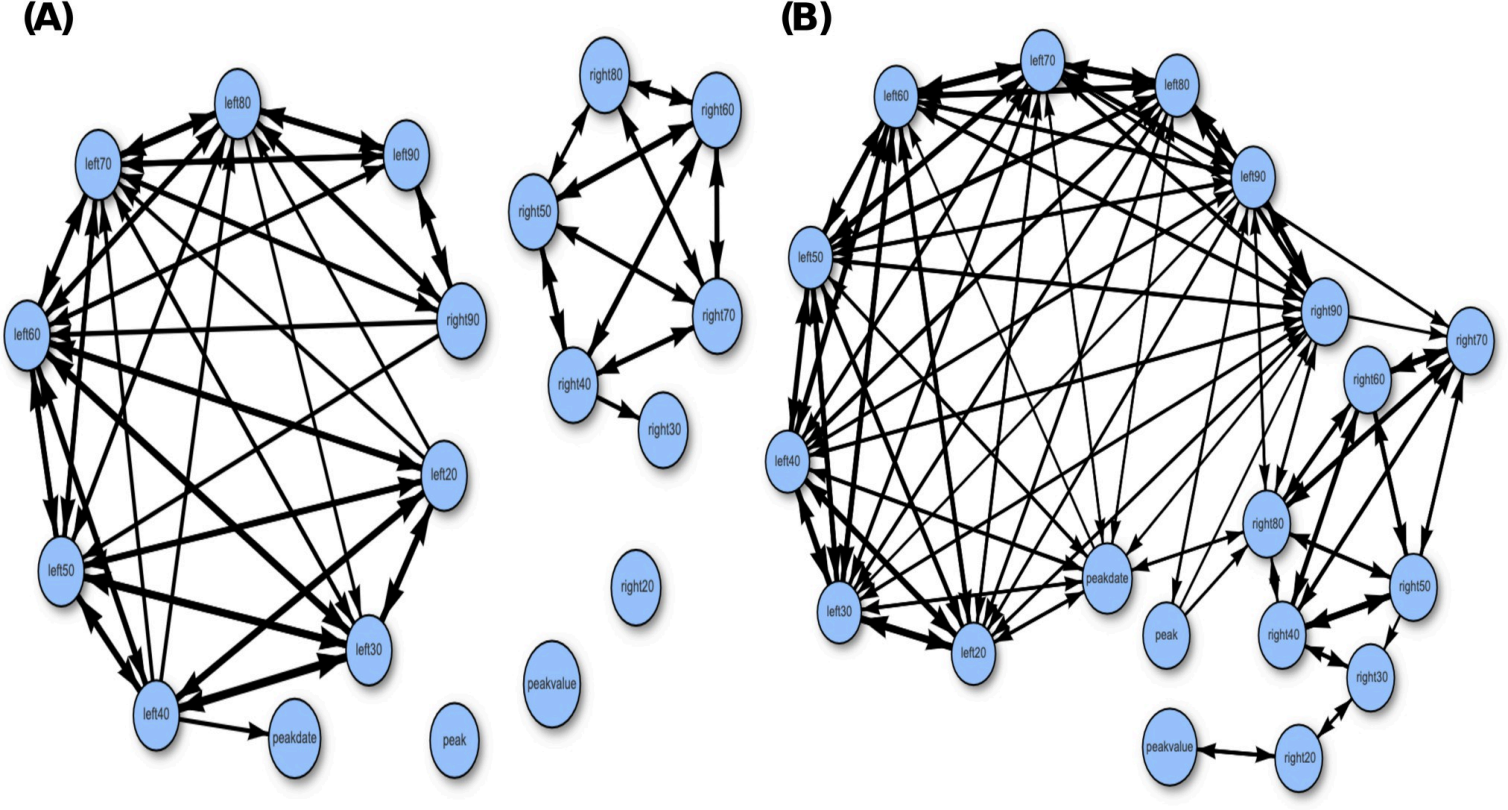
Directed associative network of all features with thresholding at: (A) 0.6; (B) 0.7.

We construct two versions of directed association networks using different threshold values, which are presented in the two panels of Fig 4. By comparing these two networks, we can observe the overall associative relationships among the 18 feature variables in terms of their strength. In panel (A), the feature variables on the left side exhibit mutual associations, while the feature variables on the right side do not demonstrate strong mutual associations. The network in panel (A) consists of two separate cliques. Although these cliques become connected in panel (B), the two groups of feature variables still do not exhibit high mutual associations.

**Fig 5:**
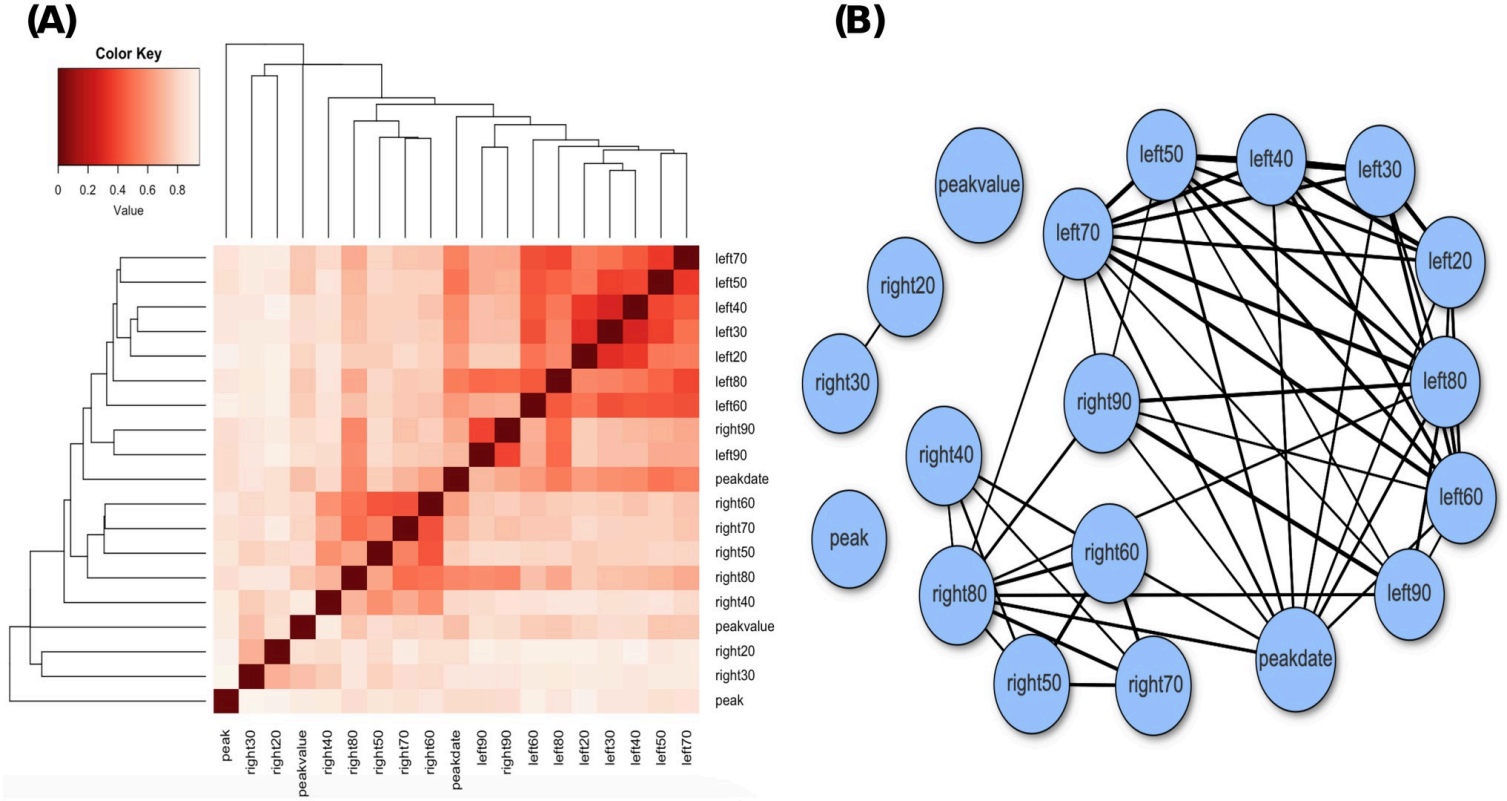
Associative heatmap (A) and network (B) of all features.

The mutual conditional entropy (MCE) is computed by averaging the two directional associations, providing a nondirectional measure of association for each pair of feature variables. Using the MCE values, we create a heatmap of the MCE matrix by rearranging rows and columns using a Hierarchical clustering algorithm. This rearrangement places similar rows or columns closer together, resulting in a clearer visual structure. The heatmap and its corresponding network are presented in Fig 5. From the heatmap, we observed a clear structure consisting of two blocks, with one block representing variables from the left side and the other block representing variables from the right side. Consistent with our previous analysis, we observe that the feature variables on the left side form a cohesive network, indicating strong mutual associations among them. However, the feature variables on the right side do not exhibit strong mutual associations in the network.

Both Figs 4 and 5 provide clear evidence of the asymmetric associations between the feature variables on the left side, which depict the growth pattern, and the feature variables on the right side, which depict the decline pattern. This observation has important implications for understanding the dynamics of infectious diseases within a closed system like Taiwan. It suggests that the constraints on the growth dynamics are more rigid compared to the constraints on the decline dynamics. This result indicates that there is limited variation in the growth patterns once the infectious disease enters a new domain. The shape of the growth pattern remains relatively fixed, with only variations in slope and peak value. In contrast, during the decline phase, there is greater freedom for the shape of the curve to vary. This shape asymmetry between growth and decline patterns suggests that behavioral impacts are also expected to be asymmetric, as we will demonstrate in the next section.

## 6 Major factors underlying dynamics of daily infection rate

In this section, we focus on computing and uncovering the major factors that contribute to two key characteristics of the daily infection rate curve: the peak value and the curvature-at-peak. The peak value serves as a measure of the intensity of Covid-19 infection within a district, while the curvature-at-peak indicates the duration during which the disease remains at its peak strength. We aim to determine whether the features related to the growth phase (Left) or the decline phase (Right) exhibit stronger associations with the peak value and curvature-at-peak. In this section, we explore these questions and present some surprising findings.

**Fig 6:**
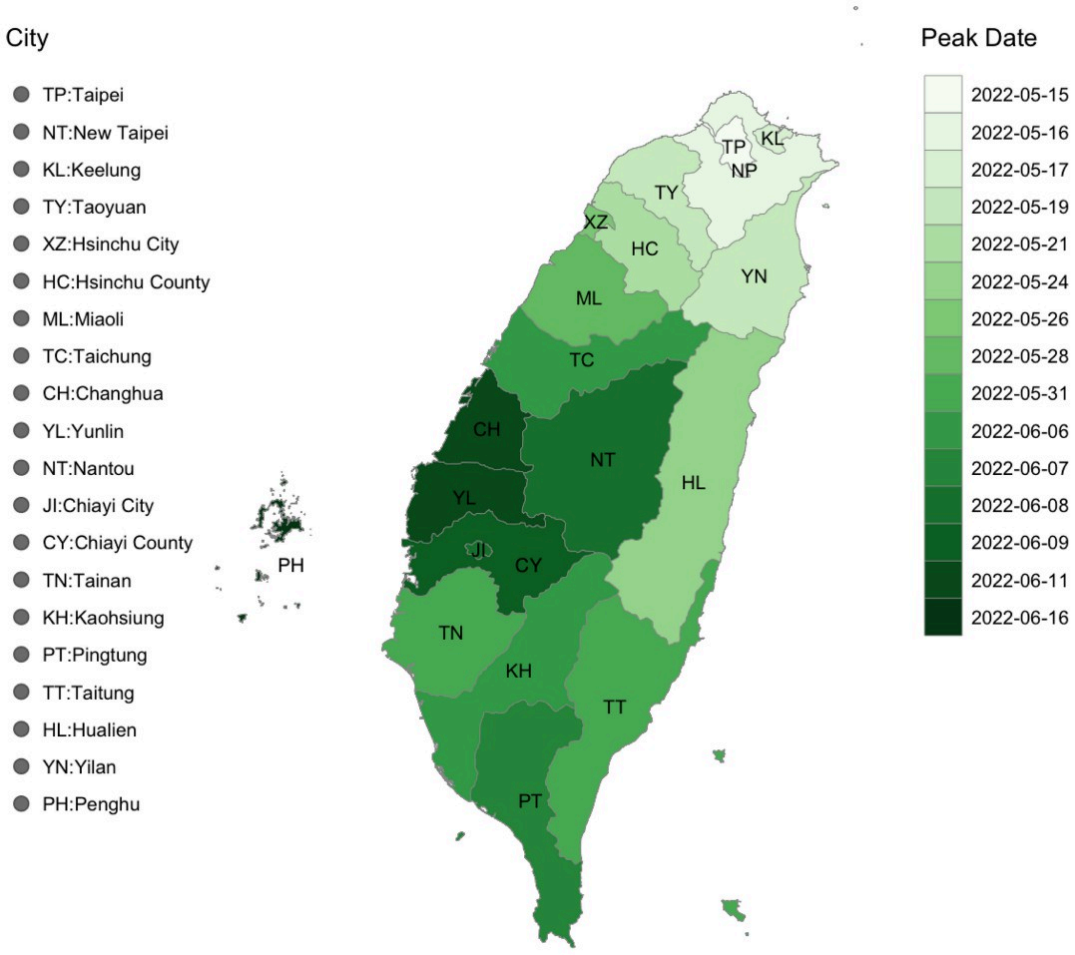
Visualization of the peak dates of all the cities in Taiwan.

Before delving into the analysis of district-level data, it is beneficial to visualize the spread of Covid-19 at the city scale in order to provide a visual context for our discussions on the dynamics of the disease. Fig 6 displays the dates of the peak infection rates across various cities in Taiwan. It is evident that the outbreak originated in Taipei (TP) and subsequently spread to its surrounding cities, including New Taipei City (NT), Keelung (KL), and Taoyuan (KY). Moving towards the west side of the island, which is densely populated, the southward spread coincides with cities that host major stations of the high-speed railway, such as Taichung (TC), Tainan (TN), and Kaohsiung (KS). The spread then extends to the suburbs of these three cities. In contrast, the east side of the island, home to smaller cities known for their tourist attractions, such as Yilan (YN) and Hualien (HL), experienced earlier instances of Covid-19 infections.

**Fig 7:**
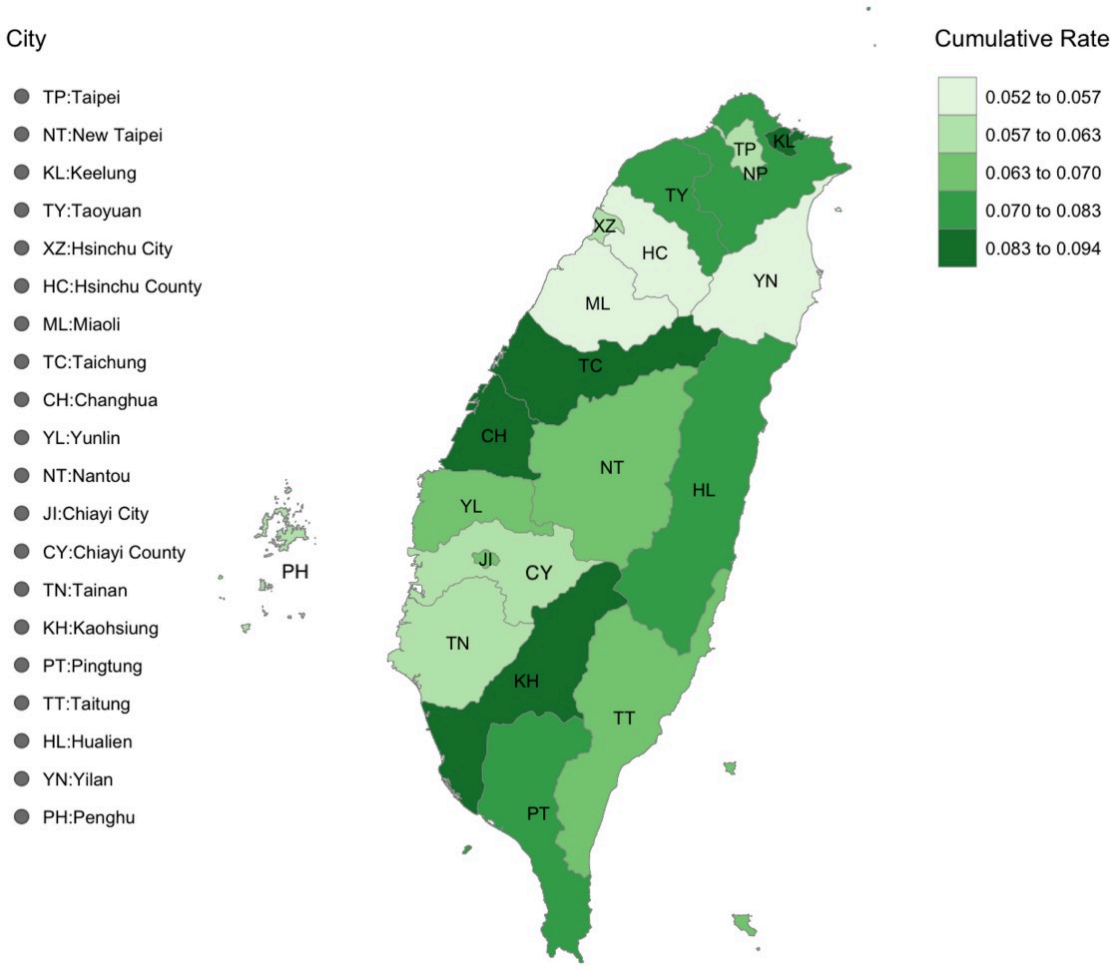
Visualization of the cumulative infection rates of all the cities at their peak dates in Taiwan.

The Fig 7 shows that Taipei has a smaller cumulative infection rate at its peak than its suburban cities of New Taipei, Keelung, and Taoyuan. Further, cities immediately south of these suburban cities are small in population size and less-densely populated. The varying cumulative rates in the three major cities: Taichung, Tainan, and Kaohsiung, are primarily due to averaging heterogeneous rates from their urban and suburban districts.

### 6.1 Exploring peakvalue

The height of the peak in the daily infection rate curve is a prominent characteristic that attracts attention. It is reasonable to assume that a district’s growth pattern plays a significant role in determining the height of its peak. Additionally, it is anticipated that the height of the peak can have a substantial impact on the subsequent decline pattern. To investigate these assumptions and expectations, we employ conditional entropy (CE) evaluations to identify the covariate features or feature sets that can influence the response variable “peakvalue”. The results of the CE calculations are summarized in Table 1, which provides insights into the factors associated with the height of the peak.

**Table 1:** Top 5 and the bottom-ranked CEs of “peakvalue” as the response variable.

1-feature	CE	SCE-drop	2-feature	CE	SCE-drop
right20	0.7202	0.4531	peakdate_right20	0.4647	0.2555
right30	0.8518	0.3215	left70_right20	0.5255	0.1947
left50	0.8707	0.3026	left20_right20	0.5261	0.1941
peakdate	0.8766	0.2967	left30_right20	0.5276	0.1926
right70	0.8816	0.2916	peakdate_right30	0.5331	0.3187
peak	1.0047	0.1686	left20_left40	0.9043	0.0407

Upon examining Table 1, we can observe the top 5 ranked features and feature pairs. The most influential 1-feature turns out to be “right20”, which corresponds to the very end of the daily infection rate curve. The relationship between “right20” and “peakvalue” can be explained as follows: a higher value of “peakvalue” indicates that it will take a larger number of days for the infection rate to decrease by 20%. The second ranked 1-feature is “right30”, which represents the declining speed at 30% of the peak. It is worth noting that “right30” is highly correlated with “right20”. The third ranked feature is “left50”, which captures the growth slope at 50% of the peak. Additionally, the fourth ranked 1-feature is “peakdate”. The association between “peakdate” and “peakvalue” reflects an important aspect of spreading dynamics: the surge in Covid-19 infection rate originated from districts in Northern Taiwan, and there is a tendency that earlier surges correspond to higher “peakvalue” values.

In the 2-feature setting, the feature pair (“peakdate”, “right20”) achieves the lowest CE, indicating that the combination of “peakdate” and “right20” provides the most informative representation of the “peakvalue” variable. It is expected to see pairs involving “right20” have low CEs, as “right20” itself has a low CE. Similarly, the feature pair (“peakdate”, “right30”) also achieves a low CE, which is in line with their individual rankings. However, it is interesting to note that the feature pair (“right20”, “right30”), despite being individually ranked first and second, does not exhibit a significantly lower CE when considered together. This suggests that the high correlation between “right20” and “right30” limits the additional information gained from their joint pair.

### 6.2 Exploring curvature-at-peak

The curvature at the robust-peak *t*_0_ plays a significant role in characterizing the smoothed curve of daily infection rate for each district. A large curvature corresponds to a small “right90” value and signifies a rapid growth followed by a steep decline. On the other hand, a small value of curvature-at-peak indicates that the district’s infection rate is sustained by the “full infection force” for an extended period centered at the robust-peak *t*_0_. This leveling-off pattern is considered unfavorable for the community within the district.

We examine the dynamics of the curvature-at-peak *t*_0_ by considering “right90” as the response variable, while excluding its close counterpart, “left90”, as a covariate feature. We present the top 5 and bottom 2 ranked CEs in both the 1-feature and 2-feature settings in Table 2. In the 1-feature setting, it is somewhat unexpected to find that the feature “left80” is ranked at the top, while “right80” is ranked fifth. There is a notable difference in the drops of their CEs. The high ranking of “left80” can be attributed to its strong association with “left90”, which is nearly equivalent to “right90”. However, what is surprising is the fifth position of the feature “right80” after “left50”. This asymmetric outcome further highlights the distinction between the growth and decline patterns in the curves of the daily infection rate.

**Table 2:** Top 5 and bottom 2 ranked CEs of “right90” as the response variable for curvature.

1-feature	CE	SCE-drop	2-feature	CE	SCE-drop
left80	0.5046	0.7138	left80_right80	0.2440	0.2606
left70	0.6386	0.5798	left80_right20	0.3071	0.1975
left60	0.6957	0.5228	left80_right70	0.3307	0.1739
left50	0.7128	0.5056	left80_right50	0.3480	0.1566
right80	0.7644	0.4540	left80_peakdate	0.3651	0.1395
peakvalue	0.9991	0.2193	peakvalue_right40	0.8287	0.1474
right30	1.1000	0.1014	right40_right30	0.9016	0.0746

In the 2-feature setting, we observe that the feature pair (“left80”, “right80”) achieves the lowest CE. However, it is noteworthy that the reduction in CE for the joint pair is smaller than the sum of the reductions for the individual features. It is expected to find that pairs involving “left80” have low CEs, but this is only the case when “left80” is paired with a “right” variable. When “left80” is paired with a “left” variable, the reduction in CE is not as significant due to the high correlations between them.

## 7 Geographic and social-economic effects

In this section, we aim to investigate whether the growth and decline patterns of the daily infection rate exhibit geographic and social-economic differences across the 79 districts considered in our study. Specifically, we focus on the top 12 districts in population for each of the six municipalities and all seven districts in Keelung. To analyze these effects, we introduce two new binary features: “North-vs-South” to capture the geographic effect and “urban-vs-suburban” to capture the social-economic effect. We will employ conditional entropy computations using these two binary features as response variables to investigate the geographic and social-economic effects on the daily infection rate patterns.

### 7.1 Exploring geographic effects

In the context of geographic effects, we classify districts into two categories: North and South, based on the cities they belong to. The North category comprises districts from Taipei (TP), New Taipei City (NT), Keelung (KL), and Taoyuan (TY). Conversely, the South category consists of districts from Taichung (TC), Tainan (TN), and Kaohsiung (KH). By employing this binary feature, we can investigate whether there are discernible variations in the growth and decline patterns between districts located in the northern and southern regions of Taiwan.

For the growth pattern analysis, we employ the K-means clustering algorithm with K = 4 to merge a set of sequentially dependent features into a single fused feature. This technique helps alleviate the curse of dimensionality. Specifically, we consider a district’s 5-dimensional vector (left30, left40, left50, left60, left70) and assign it to one of the four clusters based on its characteristics. We denote this clustering-based growth feature as “left30to70”. Additionally, we have two more growth features named “left30to50” and “left30to60”. Similarly, for the decline patterns, we introduce three fused features: “right30to50”, “right30to60”, and “right30to70”. These six fused features are investigated using our major selection protocol to examine their explanabilities on the binary response variable representing “North” or “South”. The conditional entropies (CEs) and the corresponding significant conditional entropy (SCE) drops for these features are reported in Table 3.

**Table 3:** Testing North-vs-South for geographic effect.

1-feature	CE	SCE-drop	2-feature	CE	SCE-drop
left30to70	0.27619	0.4130	left30to70_right30to70	0.1744	0.1017
left30to60	0.2955	0.3936	left30to50_right30to70	0.1819	0.1167
left30to50	0.2986	0.3905	left30to70_right30to60	0.2100	0.0661
right30to70	0.6059	0.0832	left30to60_left30to70	0.2699	0.0062
right30to60	0.6144	0.0747	right30to50_right30to70	0.5269	0.0789
right30to50	0.6300	0.0591	right30to50_right30to60	0.6104	0.0039

Based on the findings presented in Table 3, the fused growth feature “left30to70” achieves the lowest conditional entropy (CE), followed by “left30to60” and “left30to50”. Notably, the CE-drops for these growth features are five times or more significant than the CE-drops observed for the three fused decline features: “right30to70”, “right30to60”, and “right30to50”. Interpreting these CE-drops as measures of mutual information between each individual fused feature and the geographic response feature (North-vs-South), we can conclude that geographic effects are prominently reflected through growth patterns rather than decline patterns.

In the 2-feature setting, we observe that the pair of fused features (left30to70, right30to70) achieves the lowest conditional entropy (CE), indicating a strong ecological effect. Notably, the significant conditional entropy drop (SCE-drop) of 0.1017 for this pair is larger than the CE-drop of “right30to70” alone. This result provides assurance that the combined growth and decline patterns can indeed better capture the geographic effect underlying the dynamics of the Covid-19 daily infection rate. On the other hand, combinations of growth-and-growth and decline-and-decline patterns do not exhibit any significant effects beyond their individual effects.

### 7.2 Exploring social-economic effect

In the context of exploring the social-economic effect, it is important to consider the distinction between urban and suburban areas. Taipei, being the capital city of Taiwan, has several factors that contribute to its higher social-economic status compared to districts in New Taipei City, Keelung, and Taoyuan. These factors include housing prices, availability of open public spaces, and various others. As a result, we classify the 12 districts in Taipei as urban. On the other hand, the 31 districts in the surrounding cities of New Taipei City, Keelung, and Taoyuan are classified as suburban. In the case of Taichung, Tainan, and Kaohsiung, these cities include both urban and suburban districts due to recent expansions of their city administrations to incorporate districts from the surrounding counties with the same names. Therefore, for this particular example, we exclude districts from these three cities.

We utilize the same set of six fused feature variables as in the previous subsection to examine the social-economic effect. The explanabilities based on CEs are presented in Table 4. Our analysis reveals several significant findings. In the 1-feature setting, individual fused features exhibit limited urban-vs-suburban effects. However, in the 2-feature setting, we observe remarkable interactive phenomena. The pair (left30to60, right30to70) achieves an SCE-drop of 0.1504, which is more than seven times the CE-drop of “right30to70”. This effect is also evident in Table 5, where the majority of columns have very low or even zero CEs. Similar strong interactive effects are observed for the pairs (left30to70, right30to50) and (left30to50, right30to70). In contrast, pairs consisting of two growth features or two decline features do not exhibit such effects.

**Table 4:** Testing urban-vs-suburban for social-economic effect.

1-feature	CE	SCE-drop	2-feature	CE	SCE-drop
left30to60	0.6338	0.0359	left30to60_right30to70	0.4833	0.1504
left30to50	0.6387	0.0310	left30to70_right30to50	0.4864	0.1590
left30to70	0.6454	0.0243	left30to50_right30to70	0.4943	0.1444
right30to70	0.6507	0.0190	left30to60_right30to60	0.5731	0.0607
right30to50	0.6510	0.0187	left30to50_left30to70	0.6326	0.0061
right30to60	0.6616	0.0081	right30to50_right30to60	0.6503	0.0006

**Table 5:** Contingency table of (left30to60, right30to70) vs urban-vs-suburban for testing urban-vs-suburban for social-economic effect.

U-S/L_R	1-1	1-2	1-3	1-4	2-1	2-2	2-3	2-4	3-1	3-2	3-3	3-4	4-1	4-2	4-3
urban	1	1	2	1	0	1	7	7	2	4	1	0	1	1	2
suburban	0	0	2	0	9	4	11	10	0	0	5	1	2	1	3

These remarkable interactive effects of growth and decline patterns strongly suggest that curves of daily infection rates derived from urban districts are fundamentally distinct from curves derived from suburban districts. These findings align with our observations that urban curves have lower peak values and smaller curvatures-at-peak.

In summary, we have successfully examined the geographic and social-economic effects underlying the spreading dynamics of Covid-19 daily infection rates. Additionally, in this section, we have demonstrated a methodology for mitigating the curse of dimensionality using clustering algorithms.

## 8 Growth and decline similarity detailed

Based on the non-directed and directed networks illustrated in Figs 4 and 5, we observe that the feature variables belonging to the Left group are highly correlated with each other, while the feature variables in the Right group exhibit weaker associations. The associations between these two groups are also not significant, except for the pair “left90” and “right90”. Additionally, the calculated conditional entropies (CEs) for testing the geographic effect of North-vs-South indicate that the Left feature variables are more informative than the Right feature variables.

Given this understanding, we proceed to evaluate the degree of similarity among all districts and age groups based on the Left and Right feature variables separately. We employ the Euclidean distance as a similarity measure, as it allows us to consider all feature variables together. It is expected that the resulting patterns of similarity among district- or age-group-specific curves of daily infection rates will neither be completely blurred nor clearly distinct. These considerations motivate us to compute the degree of similarity based on the Left and Right feature variables separately.

### 8.1 Geographic effect via similarity among districts

In this subsection, we examine the similarity among districts within each of the seven cities. Each district is identified by a three-letter code name. The cities are represented by a two-capital-letter code, as illustrated in Figs 6 and 7. For example, “TP” represents Taipei and “NP” represents New Taipei. The two-capital-letter code is followed by a lowercase letter from “a to l,” which corresponds to one of the 12 districts within the city.

Within these 79 code-named districts, we assess the degree of similarity based on the Left feature variables using the Euclidean distance metric. To construct a clustering tree, we employ the Hierarchical clustering (HC) algorithm with the Ward-d2 module. The resulting HC-tree consists of 79 tree leaves, which are grouped and located based on their similarity. Code-named districts that share a common branch in the tree are considered to be similar to a certain degree, determined by the level of the tree where the branch is situated. For example, if two code-named leaves are grouped together in a branch at the lowermost level, it indicates a high degree of similarity between those districts. The quantification of this visible similarity can be further evaluated using the tree geometry of the HC-tree. This evaluation is performed using a binary coding scheme proposed and developed in [16].

**Fig 8:**
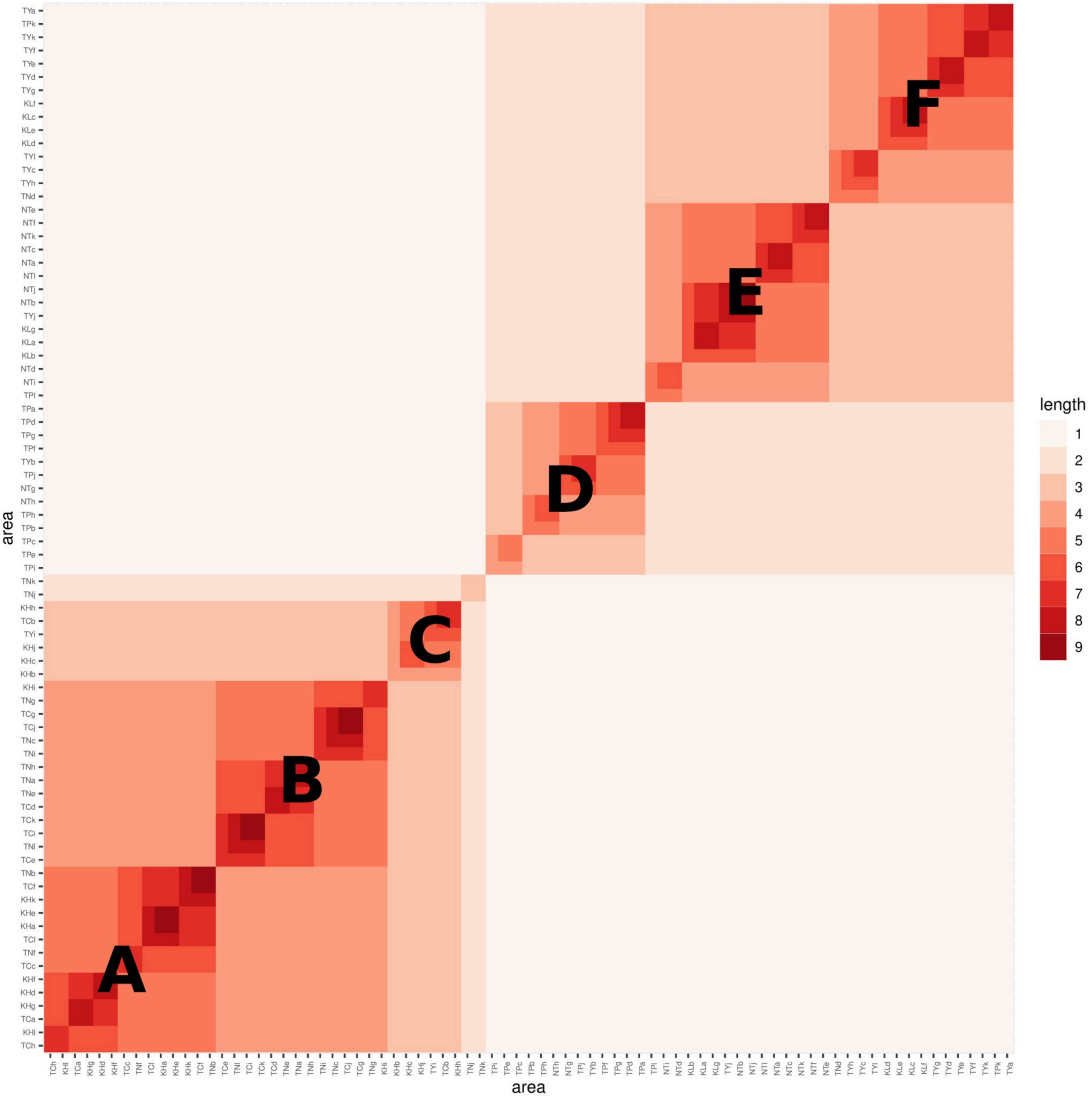
Growth similarity among 79 districts.

The HC-tree follows a binary structure, where each internal node splits into two subbranches. Hence, we can employ a simple binary coding scheme, using “left” to represent 0 and “right” to represent 1, to encode each tree leaf (or code name) from the top level to the bottom level of the tree. This binary coding scheme allows us to locate each tree leaf by utilizing a segment of binary codes. The length of the binary coding segment shared by two tree leaves directly reflects their level of similarity. The longer the common coding segment, the higher the degree of similarity between the corresponding tree leaves. We create a heatmap based on the tree-distance matrix derived from this coding scheme, as illustrated in Fig 8. The code-named districts are arranged along the row and column axes of the matrix according to the structure of the HC-tree.

The heatmap presented in Fig 8 exhibits distinct block patterns of two sizes: large and medium. There are eight medium-sized blocks denoted by capital letters from “A” to “F”. Additionally, there are two large blocks. The upper-right large block encompasses the {D, E, F} medium blocks, which correspond precisely to the districts of four cities in the northern region: Taipei (TP), New Taipei (NP), Taoyuan (TY), and Keelung (KL). Among these three medium blocks, the medium block labeled as D exclusively comprises 13 districts, including 10 districts from Taipei (TP) and 2 districts from New Taipei City (NT). All other 10 districts from New Taipei City are in the medium block E. 9 out of 12 districts of Taoyuan (TY) are in the medium block F.

On the other hand, the lower-left large block comprises three medium blocks: {A, B, C}. The majority of districts from Tainan (TN) are found in median-block-B, which also includes some districts from Taichung (TC). On the other hand, the districts in median-block-A are predominantly from Taichung (TC) and Kaohsiung (KH). This suggests that the decline patterns of districts in Taichung (TC) exhibit similarities to both Tainan (TN) and Kaohsiung (KH). However, it is worth noting that geographically, Tainan (TN) is situated between Taichung (TC) and Kaohsiung (KH).

The clear separation between the North and South regions observed in the heatmap is mainly attributed to the feature variables of the Left, which represent the growth pattern of the daily infection rate curve. These findings align with the geographic effects testing results discussed in the previous section.

**Fig 9:**
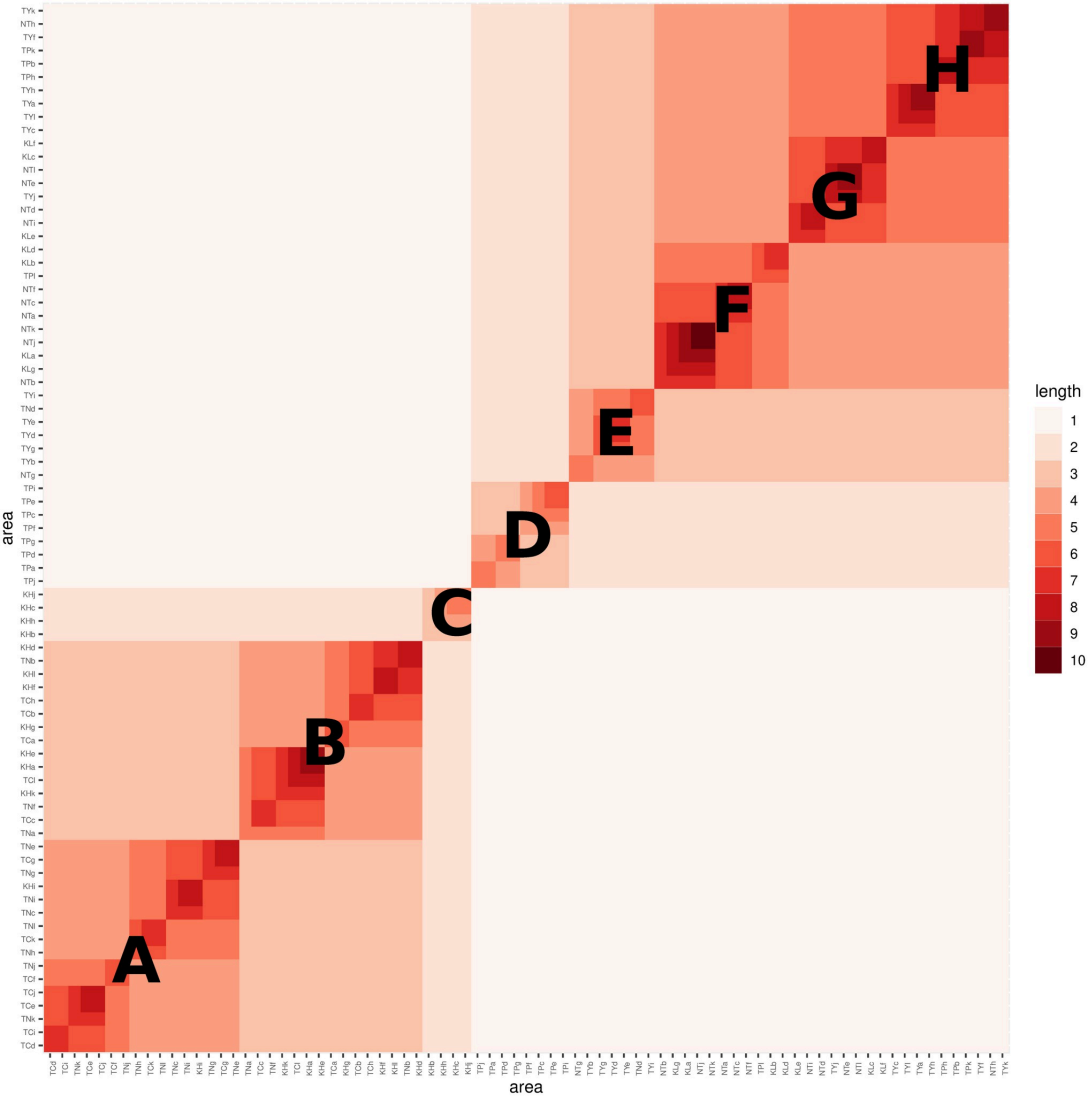
Decline similarity among 79 districts.

Similarly, we investigate the similarity among the 79 districts based on the decline perspective using the feature variables of the Right. We construct an HC-tree with 79 tree leaves representing the districts. Using the binary coding scheme, we create a tree-distance matrix of size 79 × 79, which is then visualized as a heatmap in Fig 9. Once again, we observe a clear separation between the North and South regions. The big block representing the North consists of the median blocks {D, E, F, G, H}, while the big block representing the South consists of the median blocks {A, B, C}. Among these five medium blocks, the medium block labeled as D exclusively comprises eight out of twelve districts from Taipei, while the remaining four medium blocks contain a mixed composition of districts primarily from New Taipei (NP), Taoyuan (TY), and Keelung (KL).

The smallest medium block, C, exclusively consists of four districts from Kaohsiung. However, it is interesting to observe that the medium blocks A and B do not exhibit a clear mixture of district memberships from Taichung (TC), Tainan (TN), and Kaohsiung (KH). The medium block A primarily includes districts from Tainan (TN) and some districts from Taichung (TC). In contrast, the medium block B consists of districts mainly from Taichung (TC) and Kaohsiung (KH). It is worth noting that Tainan (TN) is geographically situated between Taichung (TC) and Kaohsiung (KH).

In summary, our analysis of the growth and decline patterns reveals multiscale patterns of similarity among the 79 city-districts. These patterns are reflected in the block structures observed in the heatmaps of tree distances and shared coding segments. These findings align with the geographic effects discussed earlier. Importantly, our approach to quantifying degrees of similarity using HC-trees offers a new method for pattern recognition. It is noteworthy that the resulting pattern information is visible and interpretable, providing valuable insights into the dynamics of the Covid-19 infection rates at the district level.

### 8.2 Social-economic effect via similarity among age groups

In this subsection, we explore the social-economic effect by examining the similarity among age groups based on the curves of daily infection rates. We hypothesize that the similarity among age groups reflects the social-economic effect, as different age groups are associated with distinct social and economic factors that shape their behaviors and interactions.

For example, different school-age groups are connected to specific school districts that implement different Covid-19 regulations. Young adults and middle-aged individuals residing in different districts may commute to work using different transportation modes and frequent different locations of companies. Additionally, different senior age groups have access to various public open spaces and facilities with diverse conditions. Therefore, we anticipate that the social-economic effect, particularly in terms of urban-vs-suburban statuses, will be evident through the similarity observed among age groups.

We focus on four age groups identified using a 4th digital code: 1 for 0-19, 2 for 20-34, 3 for 35-54, and 4 for 55+. These codes are linked to the 3-letter codes representing each district. We specifically examine these age groups within the 12 districts of Taipei (TP) and the 12 districts of New Taipei City (NT). Our objective is to assess the similarity among the 96 curves representing the daily infection rates within these age groups and districts, similar to the previous subsection.

**Fig 10:**
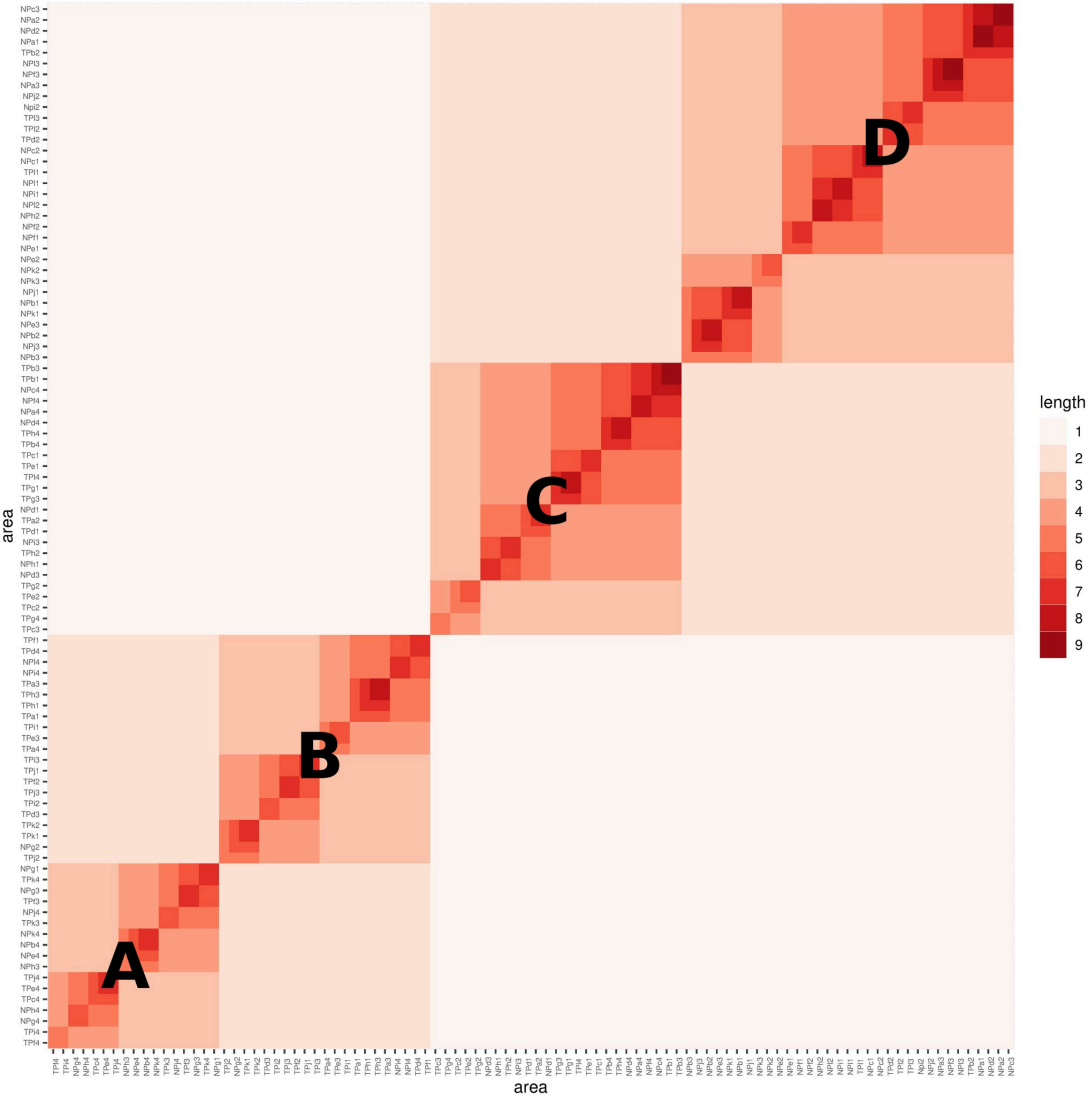
Growth similarity among age groups in two Taipei and New-Taipei cities.

The heatmap of coding segment lengths, constructed by overlaying the HC-tree with 96 leaves onto the row and column axes, reveals distinct multiscale block patterns from the growth perspective of Left features. In Fig 10, we observe visible multiscale block patterns. For simplicity, we label two median blocks within each big block with capital letters A to D. The lower-left big block shows a clear division between median-block-A and median-block-B. Median-block-B primarily consists of code-IDs representing age groups 1, 2, and 3 in Taipei (TP), while median-block-A consists of code-IDs representing age group 4 in New Taipei City (NT) and Taipei (TP). In contrast, median-block-C has a mixed composition of code-IDs in terms of age groups and TP vs. NT, while median-block-D primarily consists of code-IDs representing age groups 1, 2, and 3 in New Taipei City (NT).

**Fig 11:**
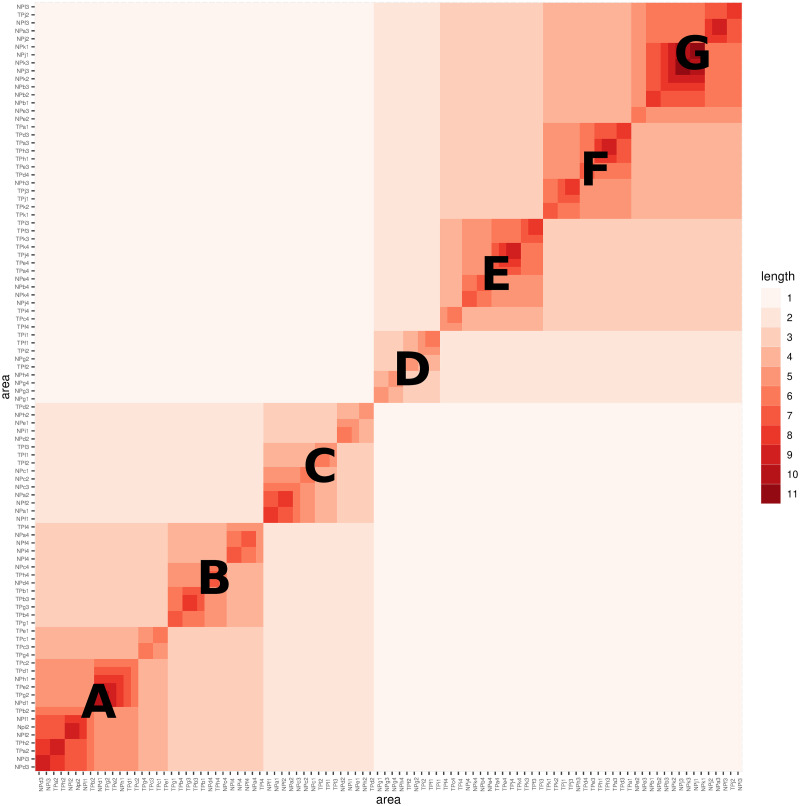
Decline similarity among age groups in two Taipei and New-Taipei cities.

Similarly, from the decline perspective of Right features, the heatmap of coding segment lengths is constructed by overlaying the HC-tree with 96 leaves onto the row and column axes, as shown in Fig 11. This heatmap exhibits clear block structures with two scales: the big-block scale in the lower-left and upper-right regions, displaying a distinct separation. In addition, we label 3 median blocks within the lower-left big block and 4 median blocks within the upper-right big block with capital letters A to G, respectively. Median-block-A consists of 15 members, primarily code IDs representing age groups 1 and 2 from districts in both Taipei (TP) and New Taipei City (NT). Similarly, median-block-C also consists of 17 members primarily representing age groups 1 and 2. On the other hand, median-block-B consists of 13 members primarily representing age group 4, as does median-block-E with its 14 members. Median-blocks F and G have a mixture of code IDs representing age groups 1, 2, and 3 from both cities.

In summary, based on our analysis of the heatmaps and the corresponding growth/decline curves, we observe a distinct separation between suburban New Taipei City (NT) and urban Taipei (TP) in terms of growth patterns among age groups. However, this separation is not as apparent when considering decline patterns. These findings highlight the influence of social-economic factors on the growth patterns among age groups and indicate that the relationship between geographic location and decline patterns may be less pronounced.

## 9 Conclusions

This paper examines the dynamics of Covid-19 infection in Taiwan, focusing on the spreading characteristics and human behavioral effects. The study period was carefully selected to capture the early stages of the outbreak when Taiwan had relatively low infection rates and was considered a controlled environment for analyzing the disease’s dynamics. By analyzing structured data from 79 curves of daily infection rates across 79 districts in 7 cities, we employ Theoretical Information measurements to identify the major factors that determine two key characteristics of the curves: peak values and curvature-at-peak. Additionally, we investigate how geographic and socio-economic factors related to human behavior influence the growth and decline patterns of the curves. To further validate our findings, we utilize the hierarchical clustering algorithm to construct tree-distance based heatmaps for both overall growth and decline patterns, as well as age-group-specific curves. These heatmaps provide an alternative approach to confirm the effects of North-vs-South and Urban-vs-Suburban factors in shaping the spread of the virus.

In summary, this paper makes several key contributions. Firstly, we successfully extract 18 features from unstructured curves of daily infection rates at two different scales: the city’s district level and the district’s age group level. This structured data format allows us to comprehensively characterize the growth and decline patterns of all curves across these two scales. The extraction of these features provides a natural and effective approach for analyzing functional data associated with the spread of the virus.

The second contribution of this paper lies in the analytical presentations of the asymmetry between the growth and decline patterns of the curves. This is achieved by employing a major factor selection protocol based on conditional entropy. By considering the “peakvalue” and “curvature-at-peak” as separate response variables, we are able to identify distinct sets of major factors that underlie the dynamics of each variable. Specifically, the “peakvalue” is found to be highly associated with features on the Left that capture the growth patterns, while the “curvature-at-peak” is strongly associated with features on the Right that characterize the decline patterns. These computational results provide quantitative insights into the asymmetry observed in the visible growth and decline patterns of the infection curves. Moreover, the major factor selection protocol demonstrates a data-driven approach to understanding complex dynamics without relying on pre-defined structures or assumptions.

The third contribution of this paper is the identification of geographic and social-economic effects through binary encodings of all districts. Specifically, we examine the North-vs-South geographic effect and the urban-vs-suburban social-economic effect. Remarkably, these effects are determined solely based on the binary encoding of districts, without the need for individual-level geographic or social-economic data. Furthermore, we validate these behavioral-oriented effects by analyzing the similarity between district-specific and age-group-specific curves, focusing on regions of growth and decline, respectively. It is worth emphasizing that our novel approach of using coding-length-based tree-distance heatmaps provides an effective visualization tool for representing hierarchical clustering trees, offering a fresh perspective on analyzing and interpreting the results.

In conclusion, our findings converge to a key take-home message: human behaviors play a significant and computable role in shaping the dynamics of Covid-19 infection spread. Through our study in Taiwan, we provide a fresh perspective on understanding not only the dynamics of Covid-19 but also the spreading dynamics of other infectious diseases. By shedding light on the impact of human behaviors, we aim to contribute to the broader field of infectious disease research and inspire further investigations in this area. Our hope is that this message from Taiwan will stimulate new avenues of inquiry and facilitate a deeper understanding of the complex interplay between human behaviors and the spread of infectious diseases.
